# Extensive necrotizing fasciitis of scrotum and abdominal wall: Report of two cases and a review of the literature

**DOI:** 10.3389/fsurg.2022.952042

**Published:** 2022-07-19

**Authors:** Zhe Meng, Yanchen Wang, Jun Chao, Yongjian Ji, Yaofei Sun, Jiang Zhu, Tongbin Gao, Si Chen, Shenyang Wang

**Affiliations:** ^1^ Yuhe Campus of Weifang Medical College, Weifang, China; ^2^ Department of Urology, Weifang people’s Hospital, Weifang, China

**Keywords:** necrotizing fasciitis, debridement, antibiotic, vacuum sealing draina(VSD), necrotizing fasciitis, debridement, antibiotic, vacuum sealing draina(VSD)

## Abstract

The incidence rate of necrotizing fasciitis(NF) is low, but it has a high mortality rate. At present, it lacks experience in clinical treatment in municipal and county-level hospitals, insufficient awareness of disease risk, lack of experience in disease surgical intervention, and lack of a set of mature treatment norms and standards. Most patients have no time to transfer to a higher hospital for treatment. In January and April 2022, two cases of large-scale necrotizing fasciitis of the scrotum and abdominal wall were treated in the Department of Urology of Weifang people's Hospital respectively and were clinically cured after active surgical debridement combined with broad-spectrum antibiotics. Through the retrospective analysis of the diagnosis and treatment of two cases of necrotizing fasciitis, this paper analyzes and summarizes the scope of surgical debridement of NF, postoperative dressing changing skills, timing of multiple debridements, application and timing of vacuum sealing drainage(VSD), and the combined use of antibiotics. To provide experience for clinical diagnosis and treatment of necrotizing fasciitis.

## Introduction

NF is an infectious disease with pain, fever, large areas of red and swollen skin, and unclear boundaries, which is commonly seen in the perianal area, limbs, and abdomen. In the progress of the disease, there may be black purple necrosis of the skin, malodorous pus flows out after ulceration (1). The patient has obvious systemic poisoning symptoms, easy to be complicated with sepsis, and even septic shock, which is life-threatening. The common pathogenic bacteria of the disease are mixed infections of a variety of bacteria. The extensive synergistic effect of aerobic bacteria and anaerobic bacteria makes the disease complex and rapid progress, which is also the reason why the infected area often has a foul odor. People with low immunity, combined with diabetes, liver disease, a history of radiotherapy, and chemotherapy for malignant tumors, AIDS, and other diseases are the susceptible population.

The great difference in the reported mortality of necrotizing fasciitis is that the prognosis of the disease is closely related to the delay of surgical intervention, so early diagnosis and treatment are the keys, and intraoperative diagnosis is also a choice when the diagnosis is difficult. There has been a comprehensive and unified standard for the diagnosis of necrotizing fasciitis in various studies. After active comprehensive treatment, NF still has a high mortality. Goh et al reported that the mortality rate is 21.5% (2). There is no doubt that the mortality rate of NF without surgical debridement is close to 100%, and the median mortality rate after active treatment is 32.2% (3). Moreover, the treatment process is expensive and the patients suffer a lot. This report aims at how to standardize the treatment of patients once confirmed or suspected. To reduce the hospitalization expenses and alleviate the pain of patients.

Two cases of large-scale NF in the scrotum and abdominal wall were seriously infected, and good therapeutic effects were achieved through systematic treatment. Now the two cases are introduced as follows, and further literature review is carried out to improve the diagnosis and treatment level of NF.

The family members are informed of the patient’s situation every day, and participate in the discussion of all the next treatment plans, including surgery and non-surgery. We have obtained the informed consent of the patient and their family members before writing this case.

## Patient information

### Patient 1

A 52-year-old male patient was hospitalized on April 8, 2022, due to “redness, swelling, heat, and pain in the right testicle and groin area for 8 days”. Physical examination: acute febrile symptoms, temperature 39.4°C, poor spirit, red, swollen, and hard skin in the right scrotum and groin area, high skin temperature, involving up to close to the lower edge of the right rib, forward to the outer edge of rectus abdominal muscle, and backward to the posterior axillary line. The scrotum is swollen, the lower part is broken, and the foul-smelling pus flows out. Admission blood glucose: 17.7 mmol/L; Blood routine WBC 12.07*10^9^/L, GRAN 92.1%, GRAN 11.11*10^9^/L, rapid C-reactive protein 219.3 mg/L, procalcitonin 14.300 µg/L.

The initial diagnosis was NF of the right abdomen and scrotum; Septic shock; Electrolyte disorder; Abnormal coagulation function; Hypoproteinemia; Type 2 diabetes. The scrotal skin and subcutaneous necrotic tissue excision and debridement + abdominal wall debridement were performed in the emergency on the day of hospitalization (No. 8). During the operation, the wound was expanded longitudinally from the right scrotum to the anterior superior iliac spine, the necrotic tissue was removed, washed repeatedly with hydrogen peroxide and normal saline, and filled with oil gauze (Figure 2). The necrotic tissue and pus were collected and cultured during the operation. The patient developed septic shock during the operation and was closely monitored and treated in ICU after the operation. Debridement was performed again on the 12th. The incision was expanded and debridement was performed again (Figure 3). On the 18th, debridement was performed again, the scrotal incision was sutured, and the VSD technique was used in the lower abdominal incision (Figure 4). Wound suture an skin grafting were performed on the 26th. During the operation, local granulation tissue grew well without signs of infection. The wound suture was combined with VSD again (Figure 5). After the operation, the patients were monitored and treated in our ward.

**Figure 1:**
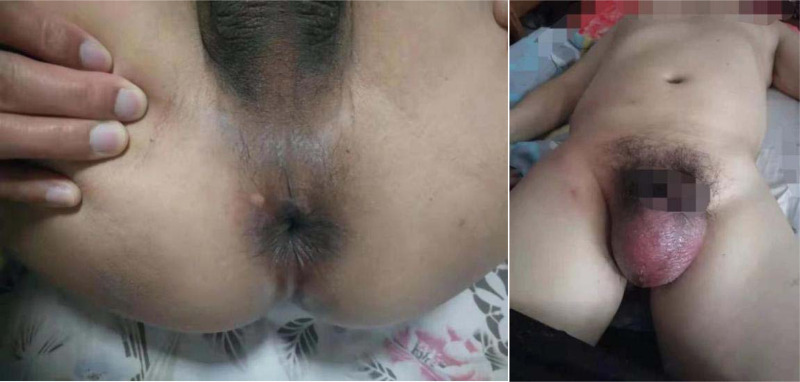
Initial abscess of patient 1.

**Figure 2:**
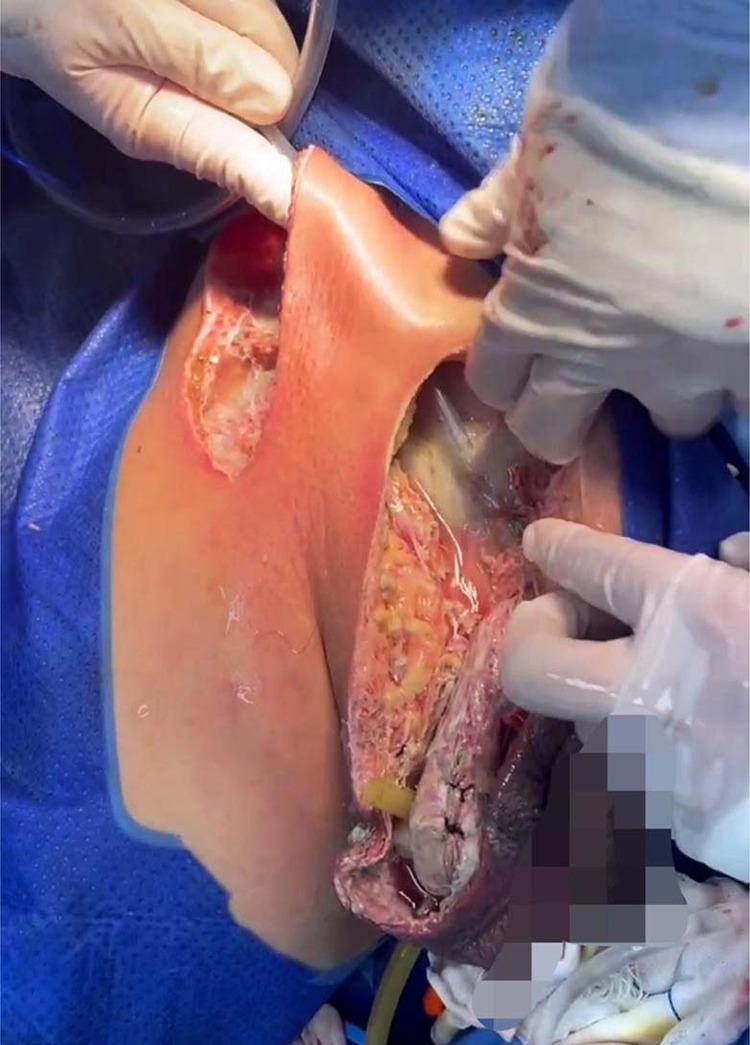
Situation during the first debridement.

**Figure 3:**
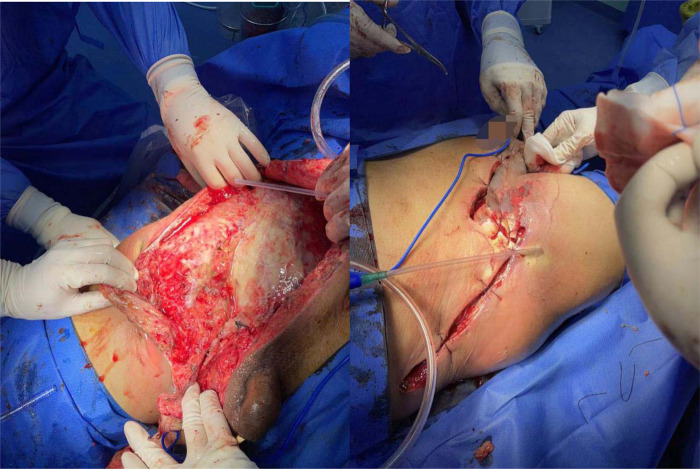
In secondary debridement, expand the incision and turn over the skin to both sides.

**Figure 4:**
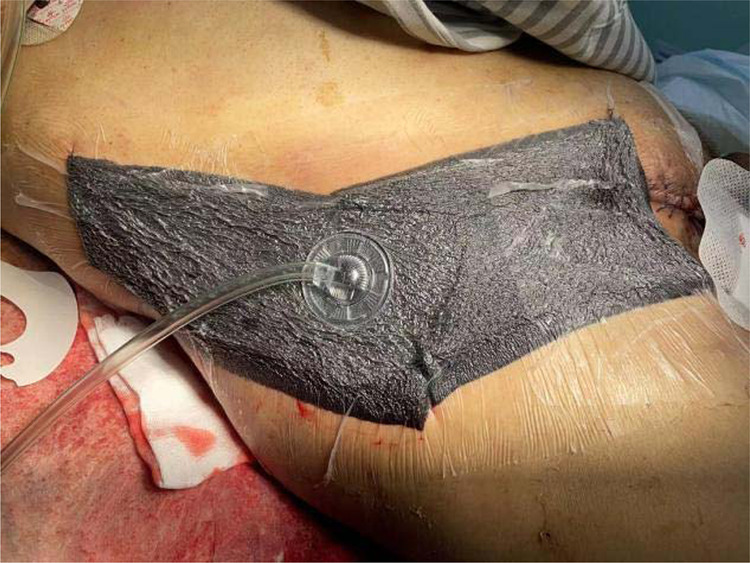
VSD after three debridements.

**Figure 5:**
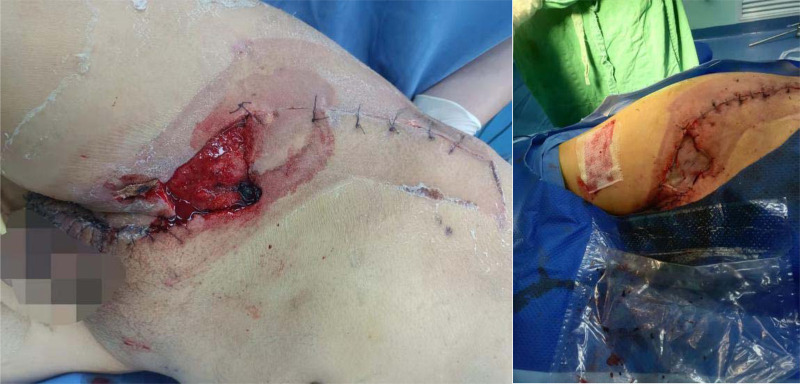
Four operations to remove VSD (left); suture and skin grafting (right).

The patient has a high fever of 38.9 degrees Celsius and obvious systemic symptoms (heart rate is 112 times/min faster and blood pressure is 96/65 mmhg lower). After the first debridement, the patient was empirically treated with penicillin (4 million u IV q6h), vancomycin (1 g infusion pump pumped into bid), and clindamycin (0.6 g IV q6h). Two days after the first operation, the dressing still has a foul odor, and ornidazole (0.5 g IV bid) was added to the treatment. Results of drug sensitivity test: Escherichia coli and Streptococcus pharyngitis. According to the drug sensitivity test adjustment, the antibiotics are vancomycin (1 g infusion pump pumped into bid), biapenem (0.6 g IV Bid), and ornidazole (0.5 g IV Bid). The patient was clinically cured and discharged after comprehensive treatment. The total length of stay was 38 days.

### Patient 2

**Figure 6:**
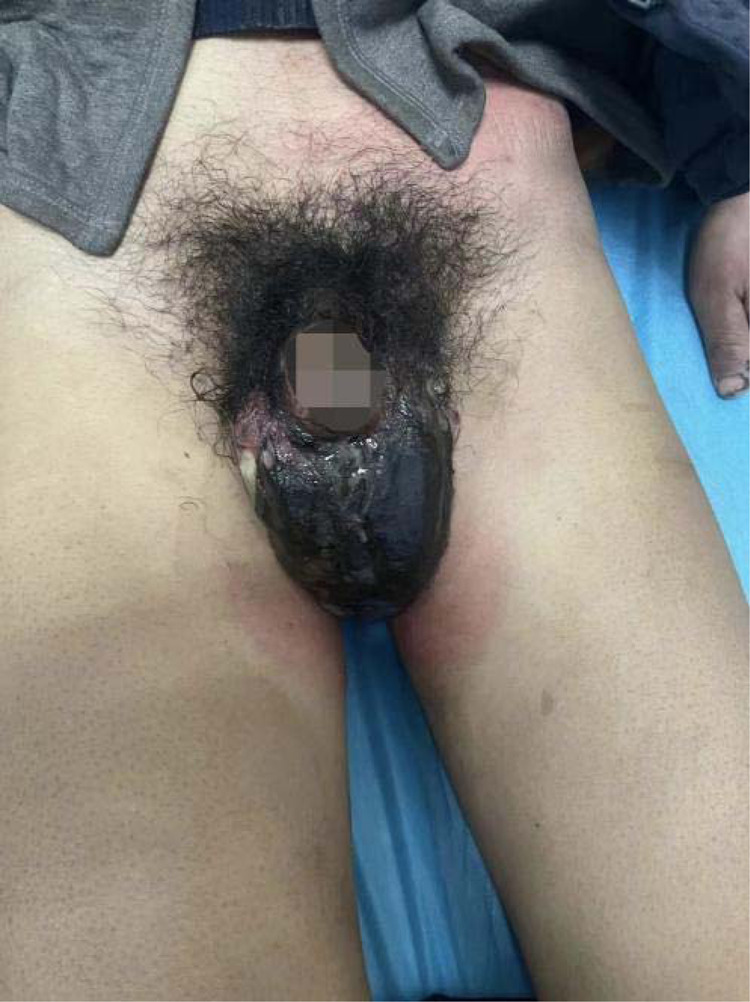
Scrotal gangrene at the admission of patient 2.

A 34-year-old male patient was hospitalized on January 21, 2022, due to a “fester of the scrotum after trauma for 5 days”. Physical examination: the left lower abdominal wall is red, swelling, and tenderness, most of the scrotal skin is black, ulcerated, necrotic, and smelly, and the left testicular sheath is swollen and exposed (Figure 6). Admission blood routine: WBC 19.49 *10^9^/L, Gran 91.1%, Gran 17.76*10^9^/L.

**Figure 7:**
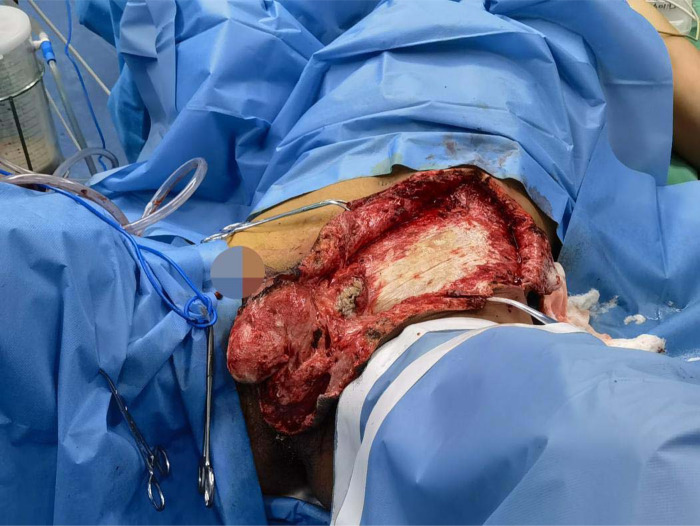
Patient 2, during secondary debridement.

The initial diagnosis was scrotal gangrene; NF in the left lower abdomen. The excision and debridement of subcutaneous necrotic tissue + Excision of left testis and epididymis were performed in the emergency on the day of hospitalization (No. 21). During the operation, the left testis and epididymis were removed, and continued to expand the incision to the left upper abdominal wall beyond the normal tissue. The necrotic tissue was removed, and the abdominal infection area was washed repeatedly with hydrogen peroxide and normal saline and filled with oil gauze. The necrotic tissue and pus were collected and cultured during the operation. On the 23rd, debridement was performed again. The Y-shaped enlarged incision was made during the operation and debridement was performed again (Figure 7). The patient was monitored and treated in our ward after the operation.

The fecal odor in the infected area of the patient is obvious, and the negative bacilli infection is mainly considered. After the first debridement, the patient empirically used piperacillin sodium and tazobactam sodium (4.5 g IV Q8H), metronidazole (0.5 g IV Q8H), and clindamycin (0.6 g IV q6h) triple antibiotics to resist infection. Drug sensitivity test results: Escherichia coli. Continue to use triple antibiotics. After comprehensive treatment, the patient's infection was controlled and his condition improved. The patients were transferred to burn surgery for further wound treatment and skin grafting. The total length of stay was 32 days.

## Discussion

NF is an acute necrotizing soft tissue infectious disease caused by bacteria invading subcutaneous tissue and fascia. The initial stage of the disease is relatively hidden, often manifested as local general inflammation, but the condition is dangerous and develops rapidly. The clinical manifestations are mainly local redness and pain, numbness after pain, black skin with bloody blisters, and malodorous purulent blood seepage. Most patients died of septic shock and multiple organ dysfunction caused by sepsis.

Early detection, diagnosis, and treatment are the keys to the cure of NF. The application of broad-spectrum antibiotics before the results of the drug sensitivity test is necessary, but it can not replace surgical debridement. While actively anti-shock, the physicians should actively control symptoms of shock, infection, and acid-base imbalance, strengthen nutritional support, pay attention to infection indicators, and beware of septic shock.

Based on previous studies, the early imaging diagnosis of necrotizing fasciitis was mostly negative. Only 24·8 percent of patients occurred gas in the soft tissue on X-ray. So the diagnosis mainly depends on the symptoms and signs (Table 1) (4). The top three early presenting clinical features were: swelling (80·8 percent), pain (79·0 percent), and erythema (70·7 percent). These are non-specific features. Clinical features that helped early diagnosis were: pain out of proportion to the physical findings; failure to improve despite broad-spectrum antibiotics; the presence of bullae in the skin. Because of the masking effect of NSAIDs, steroids, and antibiotics, the absence of pyrexia should not be used to rule out NF (2). More than half of the etiology of NF is diabetes (6, 7), and diabetes patients are often complicated with multiple bacterial infections, with a 69.2% delay in diagnosis. The incidence of various complications is high, occurs early, and the risk of death is 9 times that of non-diabetes patients (8). Patients with swelling, pain and erythema caused by skin infection and a history of diabetes or liver disease need to be highly suspected of NF (2). The best way to diagnose NF seems to be the “fingers Test”(A minimally invasive method of surgical exploration) (6).

**Table 1:** Necrotizing fasciitis signs and symptoms.

Early	Late
Local	
Skin puncture or injury	Hematic/gas bullae
Erythema	Necrosis
Warmness	Purple/blue skin color
Tenderness	Crepitus
Myalgia	Hypoesthesia
Hypersensitive skin	Sensory/motor deficit
Systemic	
Pain out of proportion	Fever (sometimes hypothermia)
Swelling	Hypotension
Fever	Mental confusion
	Multiorgan failure

Modified from Wang et al. (5).

Of the two patients reported in this article, one was a patient with diabetes. Although the diagnosis was late, the prognosis was good after active surgical debridement combined with antibiotics and VSD. The other patient with post-traumatic NF also achieved good outcomes after systematic treatment. The literature was reviewed and the following experiences were summarized.

### Surgical debridement and wound management

Early debridement of NF can significantly reduce mortality. Delayed surgical intervention and incomplete debridement lead to a significant increase in mortality (9). The time of the first debridement is directly related to the prognosis of patients (6, 10, 11). It is recommended to perform debridement within 24 h after the visit, and debridement must be thorough. The incision crosses the infected area until the normal tissue, to completely remove the pus and necrotic tissue, including the edge of the normal tissue, to prevent the rapid progress of the disease and reduce the number of unnecessary operations (12). It is recommended to make a Y-shaped or cross-shaped incision during the operation, without suture, and fully expose and open the lesion area, which is conducive to the elimination of stubborn anaerobic bacteria, to prevent the resurgence of anaerobic bacteria.

At present, there is little research data on the management of postoperative wounds and the choice of re-debridement time. Some literature recommend debridement every 12–36 h (13, 14). Although it can dynamically understand the infection, frequent and blind debridement not only increases the operational risk but also increases the hospitalization cost of patients. Reasonable postoperative wound management can reduce the number of unnecessary operations: first, the lesion area is thoroughly debridement, the knife-edge is opened, and the dressing covered by the knife-edge is wet to dry from the inside to the outside (oil yarn, wet dressing, dry dressing). Silver alginate dressing can be used as appropriate. It can inhibit the growth of microorganisms and keep the environment clean for wound bed preparation. Although silver dressing seems to be expensive, it can reduce the pain of patients and is conducive to wound recovery (15). Before infection control, the dressing should be changed at least once a day, to pay attention to the changes of local infection in time. After the fresh granulation tissue begins to grow, the number of dressing changes can be reduced as appropriate. Due to the characteristics of the infectious focus of NF, routine wound disinfection is difficult to deal with the edge of subcutaneous infection. The ideal debridement effect can not be achieved while washing with a large amount of disinfectant, and the patients suffer great pain. Therefore, the author recommends an infusion set to assist in wound disinfection and debridement. During debridement and flushing, you can cut open the disposable normal saline infusion bag (or use a glass bottle infusion set), pour disinfectant into it, and hold the edge of the infusion set with your fingers while exploring and flushing. In this way, the debridement is not only complete but also saves disinfectant water. For the selection of disinfection water, diluted iodophor water and normal saline should be used routinely, but hydrogen peroxide should be added as appropriate for patients without anaerobic bacteria control.

**Figure 8:**
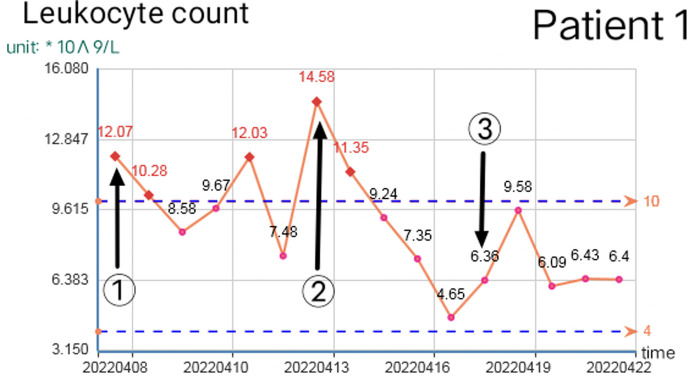
Change of leukocyte count in patient 1 (①, ②, and ③ are the debridement time of the first, second, and third operations respectively).

**Figure 9:**
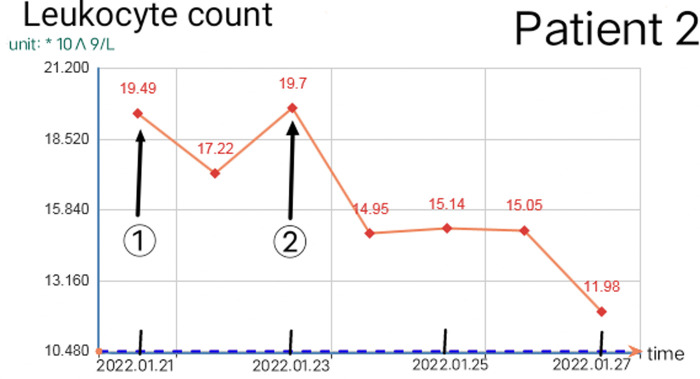
Change of leukocyte count in patient 2 (① and ② are the debridement time of the first and second operation respectively).

Reasonable postoperative wound management can reduce the number of operations, but debridement is necessary in some cases. When there are two or more of the following situations, consider debridement again: 1. The debridement of the previous operation was not complete and did not completely cross the infected area. 2. The infected area expanded significantly again, beyond the scope of the previous debridement, or a large number of local tissue necrosis. 3. The odor in the local infection area of the patient increased significantly. 4. On the premise of applying sufficient effective antibiotics, the blood routine leukocyte count was higher than normal for two consecutive times and continued to rise. 5. The patient had early manifestations of septic shock. The increase in leukocyte count and C-reactive protein is closely related to the disease (8). The increase of leukocytes is often accompanied by other indicators at the same time, which should be paid attention to. Once the white blood cell count exceeds the normal value and continues to rise, the debridement and exploration must be performed again even if there is no sign of aggravation of local infection. Because the spread area of NF infection is usually much larger than that seen in simple physical examination (9), sufficient debridement can significantly reduce the leukocyte count within 12 h, but it will rebound again before the infection is controlled. If there is no indication of local infection and aggravation of systemic poisoning symptoms before it exceeds 10*10^9^/L, close observation and dressing change can be made as appropriate. Figures 8, 9 show the relationship between debridement timing and leukocyte count in these two patients.

Once the infection is controlled, effective VSD technology is a common method for large-scale tissue defect repair. The use of VSD generally lasts for 7–10 days, several authors have changed the VAC dressing every 3e5 days (16), which should be implemented based on thorough surgical debridement and comprehensive control of anaerobic bacteria. It can reduce bacterial invasion, reduce inflammatory mediators and lactic acid accumulation, avoid residual ineffective cavities, create a good microenvironment for wound healing, promote the growth of granulation tissue and shorten the recovery time of wound healing (17–20). Premature use of VSD will lead to blockage of necrotic tissue, accumulation of exudate, aggravation of infection, and active bleeding of the wound surface, etc. More importantly, the vacuum environment provided by VSD will aggravate the anaerobic infection.

The best time to apply VSD technology should be 1. The necrotic tissue in the wound cavity should be removed. 2. The infection has passed the acute stage and has been controlled. 3. Anaerobic bacteria have been controlled (negative pus culture, negative blood culture, no odor at local infection). 4. Granulation tissue has begun to grow (8, 21, 22).

In the future, it is necessary to further standardize treatment, balance debridement, and dressing change, and maximize benefits. Avoid the high cost of over-operation and the impact of the delayed operation on prognosis. Avoid frequent dressing changes that may affect wound healing and too few dressing changes that may delay the condition of the wound.

### Combined application of antibiotics

NF disease progresses rapidly. Pathogenic bacteria usually involve a wide range of aerobic and anaerobic bacteria. The most common pathogenic bacteria are Bacteroides, aerobic Streptococcus, Enterococcus, Escherichia coli, and other gram-negative bacillus (23). Therefore, the empirical treatment plan should cover at least two antibiotics sensitive to gram-positive cocci, gram-negative bacilli, and various anaerobic bacteria, and further treatment should be guided by the drug sensitivity test results obtained from the first debridement. Necrotic tissue and pus culture should be taken during each operation to diagnose and adjust antibiotics.

The diverse characteristics of NF infection bacteria determine that antibiotics should cover a wide range. First, Streptococcus should be covered, penicillins are necessary, and broad-spectrum semi-synthetic drugs can also be selected *β*-Lactam antibiotic piperacillin-tazobactam (If the patient is allergic to penicillins, the second and third generations of cephalosporins can be selected). Secondly, anaerobic bacteria should be covered, and nitroimidazole antibiotics (such as metronidazole and ornidazole) or lincosamide antibiotics (clindamycin) can be added to kill anaerobic bacteria in time; If the patient's infected area emits a foul smell, they can be used in combination when anaerobic bacteria infection is serious. In addition, the choice of antibiotics should also cover methicillin-resistant Staphylococcus aureus (vancomycin, clindamycin, or linezolid). Carbapenems, such as meropenem and biapenem, are added when the infection range is wide and the sensitive antibiotics control is poor.

The author recommends several empirical treatment schemes of antibiotics: (1) the combination of penicillin, ornidazole, and clindamycin. (2) Combination of piperacillin, tazobactam, linezolid, and meropenem (3) Combination of ceftriaxone, clindamycin, and biapenem (23, 24). Further treatment should be carried out according to the cultural results in time. Vancomycin, as a powerful narrow-spectrum antibiotic, should be stopped in time if it loses its indications, and should be stopped before the last debridement and complete suture. Before there are no culture results, it is impossible to ensure sufficient effective antibiotics, and hormone drugs should be used with caution.

## Conclusion

NF, the infection progresses rapidly, and delayed diagnosis and treatment will have serious consequences. Early diagnosis and active surgical debridement supplemented by sensitive antibiotics are the keys to treatment (25). Reasonable wound management can reduce unnecessary debridement, reduce hospitalization costs, and alleviate patients’ pain. Close postoperative nursing and high protein energy nutritional support escort the cure and recovery of patients.

## Data Availability

The original contributions presented in the study are included in the article/Suplementary Material, further inquiries can be directed to the corresponding author/s.
